# To Catch a Fly: Landing and Capture of *Ceratitis capitata* in a Jackson Trap with and without an Insecticide

**DOI:** 10.1371/journal.pone.0149869

**Published:** 2016-02-26

**Authors:** Nicholas C. Manoukis

**Affiliations:** US Department of Agriculture-Agricultural Research Service, Daniel K. Inouye US Pacific Basin Agricultural Research Center, Hilo, Hawaii, United States of America; University of Thessaly, GREECE

## Abstract

Attractant-based traps are a cornerstone of detection, delimitation and eradication programs for pests such as tephritid fruit flies. The ideal trap and lure combination has high attraction (it brings insects to the trap from a distance) and high capture efficiency (it has a high probability of capturing the insect once it arrives at the trap). We examined the effect of an insecticide (DDVP) in combination with a pheromone lure (trimedlure) on capture of *Ceratitis capitata* using 1) digital images of surfaces of a Jackson trap analyzed via computer vision, and 2) counts of the number of flies caught in the trap and in the area under the trap. Our results indicate no significant difference in trap capture without or with insecticide (means ± SD = 324 ±135 and 356 ±108, respectively). However, significantly more dead flies were found around the trap with insecticide (92 ±53 with insecticide compared with 35 ±22 without), suggesting a possible decrease in trap efficiency due to mortality before insects enter the trap. Indeed, the average number of flies detected on all surfaces of the traps with insecticide was lower than that for lure-only (4.15±0.39 vs 8.30±1.18), and both were higher than control (no lure: 0.76 ±0.08). We found that the majority of fly sightings, 71% of the total, occurred on the inside panels of the lure-only traps, suggesting that increased efficiency of the Jackson trap may be obtained by adding a contact insecticide to those surfaces.

## Introduction

Attractant-based traps, particularly those baited with pheromone lures, are critical components of pest detection, delimitation and eradication programs worldwide [1, 2]. Examples of trap networks comprising thousands or tens of thousands of traps over a wide area include a delimitation network for *Popillia japonica* in Missouri USA [3] and a large program for *Lymantria dispar* eradication in New Zealand [4]. Use of pheromone-baited traps in a detection context is particularly common in the case of tephritid fruit flies, which are tropical pests of major concern in many parts of the world [5–7]. In California USA, for example, government agencies operate a network of over 63,000 attractant-based traps for tephritids [8]. While many factors are involved in optimizing a trapping program, including material costs, labor costs, and trap placement, it is generally agreed that detection ability increases with increasing trap attraction [9–11].

Attraction of a trap is often discussed in terms of the distance over which an insect can be brought into the trap and captured, usually using concepts such as the sampling range, attraction range and the effective attraction range [10, 12, 13]. It is often important to decouple the role of the lure in bringing the insect to the trap from the probability of the insect being captured by the trap, which is often highly contingent on the trap design (e.g. [14]). Other factors, such as the use of an insecticide, could also have an impact on the probability of capturing an insect with a trap.

The practice of using attractants to lure and trap *Ceratitis capitata* (“Mediterranean fruit fly”, or “Medfly”), one of the most important tephritid pests, is over 100 years old [15]. From at least the mid-20^*th*^ century, tert-butyl 4(and 5)-chloro-cis- and trans-2-methylcyclohexane-1-carboxylate (trimedlure) has been combined with dimethyl 2, 2-dichlorovinyl phosphate (DDVP) to kill Medflies brought into a trap and so increase the “capture efficiency” of the trap [16]. In this context, increased efficiency is an increase in the proportion of insects approaching a trap that are captured in the trap [17]. In the particular case of attractant based trapping, three factors play important roles in the capture efficiency of a trap: 1) the design of the trap itself, 2) the attractant being used and 3) the inclusion or exclusion of a non-attractive insecticide. Here, we are focused primarily on the third factor.

Adding an insecticide to an attractant-based trap can reduce trap catch, depending on the type of insecticide used (e.g. [18, 19]). If captures are lower in an attractant-based trap with insecticide compared with one without, two possible causes are usually suggested: there is a repellent effect of the insecticide (often cited for DDVP), and/or trap capture efficiency is decreased by targeted insects’ death before they can enter the trap, resulting in a non-capture [20–22].

In this study we measured if there was a difference in the alighting and capture of Medfly on Jackson traps baited with trimedlure (TML) with or without DDVP. The number of flies caught in the trap as well as the number found dead under the trap were recorded. The overall goal of this study to measure possible repellent action by the insecticide via fine-scale analysis on individual behavior on and around the trap.

## Materials and Methods

### Flies and Traps

**Fig 1:**
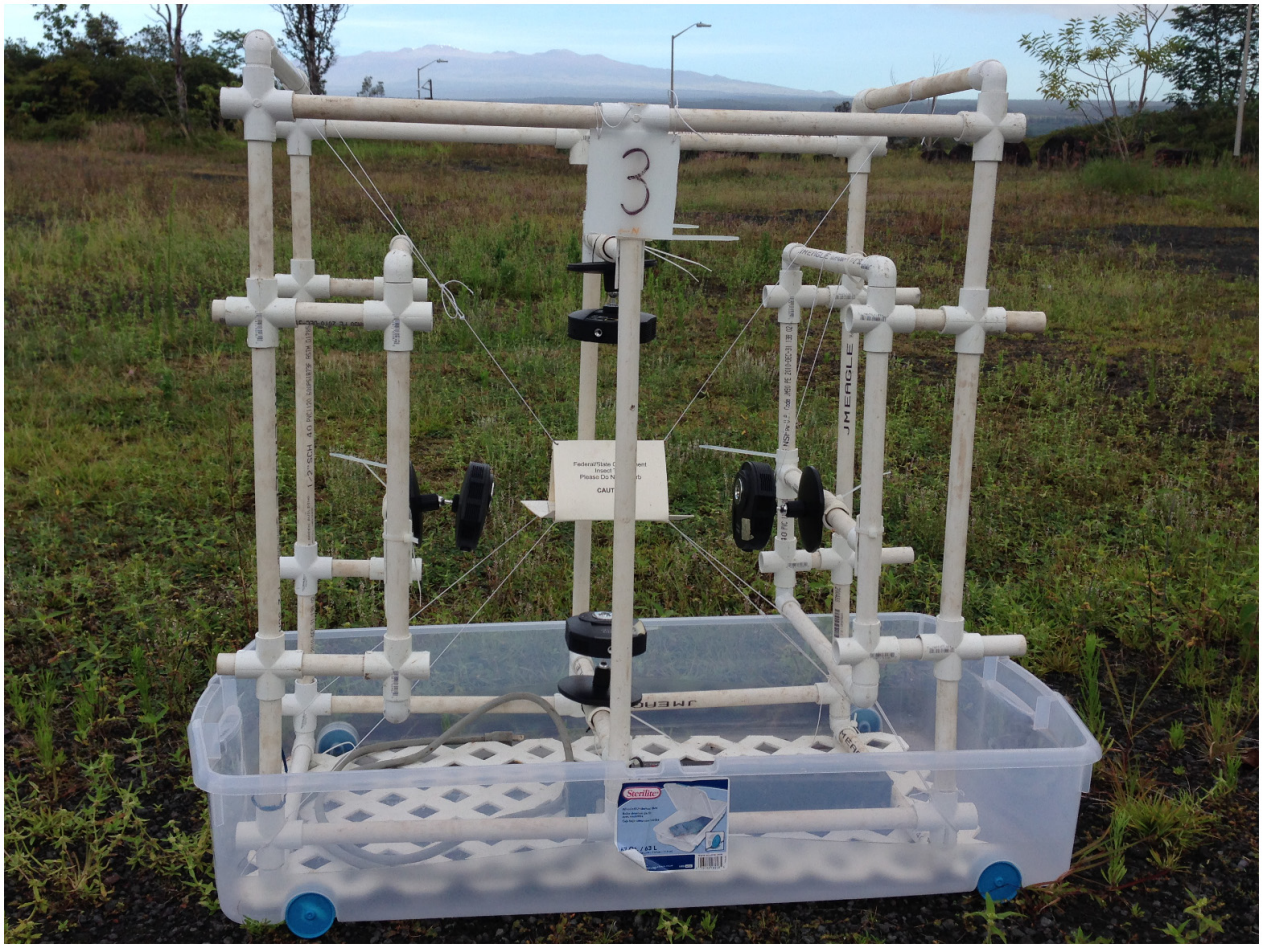
Apparatus for image capture and fly detection 5 surfaces of a Jackson trap. Four cameras were used to image two internal surfaces, both top surfaces and the bottom of the trap. We did not image the sticky panel at the bottom of the internal area. The tub used to collect flies that dies without being captured on the sticky panel is also pictured.

Experiments (seven replicates between May and July 2014) were conducted in a large screened enclosure (w x l x h = 6.1 x 15.2 x 3.0 m) at the Daniel K Inouye US Pacific Basin Agricultural Research Center (DKI-PBARC) in Hilo, Hawaii. Specific permission for this work was not required since it was conducted on USDA property and without any endangered species. The enclosure was situated in a gravel field with the nearest vegetation over 50 m away. No plants were placed in the enclosure, though there were some opportunistic grassy weeds in patches; we mowed these to keep height below 2 cm. At the start of the experiment, flies were released at the center of the enclosure. Image capture systems (Fig 1) were set in three of the four corners of the enclosure, each containing a Jackson trap that was baited with trimedlure only (“TML”), trimedlure plus a DDVP vapor strip (“TML-DDVP”) or no lure (“Control”). The corner where each treatment was placed was randomized between experimental days (replicates). Fresh DDVP was added for each replicate to the traps via Hercon VaportapeTM II strips (VT) (Hercon Enviromental, Emigsville, PA), affixed to one of the internal side panels of the trap. TML was added to treatments via a fresh 2g plastic polymer matrix plug placed at the center of the sticky panel (Scentry Biologicals, Billings, MT).

Each image capture system included four cameras (Cisco WVC 80N IP cameras, Cisco Corp., San Jose, CA) to image the two internal sides of the Jackson trap without a sticky insert (one camera for each), the two top panels (one camera) and the bottom surface of the trap (one camera). We captured 640- by 480-pixel digital images from each camera from the start of the experiments at 08:00 h until 16:30 h. The cameras were networked using 10/100 ethernet via TCP/IP protocol. Framing the images before the experiment started was possible by connecting to the cameras with a notebook computer to view the image before data gathering began. Images were copied via the network from each camera in sequence every 6 s and stored to disk by using a program written in the Python [23] programming language running on a small ARM-based computer and stored on a 64-GB USB flash drive.

*Ceratitis capitata* were obtained from the stock colony maintained at DKI-PBARC, originally derived from wild flies collected on Oahu Island around 1978. This colony has been maintained in the laboratory with occasional refreshment via the introduction of new wild material to maintain genetic diversity. It is currently reared on artificial diet [24] in approx 1m^3^ enclosures containing a total of about 50,000 flies of both sexes. Adult medflies were about 12 days old at the time of the experiments, and they were held at 26 ± 2°C and at 50 ±10% RH with a 12:12 light:dark photoperiod before being released into the field enclosure.

For each replicate we held a 1g sample of pupae from the DKI-PBARC stock colony of *C. capitata* and allowed these to emerge separately from the other pupae for the experiment (24g of pupae held for each replicate). On the day of the experiment we counted living male and female flies from the 1g sample in order to estimate the total number of males used in each experimental replicate.

Hilo is characterized by a wet tropical climate, with average rainfall around 3,220 mm per year and mean temperature between 21.9 and 24.7°C [25]. For the three months that experiments were conducted during this study the average monthly rainfall was 165mm, mean maximum daily temperature was 28°C and mean minimum daily temperature was 20°C.

At the end of the experiment we removed the imaging systems and stored them in the laboratory. Within a week we counted the number of flies caught in the sticky panel and the number of dead flies found in each tub containing the frame. We were unable to reliably differentiate males from females due to desiccation and large numbers caught in panels, so these data are for both sexes (though we expect nearly all the flies caught in the traps to be males, as trimedlure is a male attractant [26]).

### Automatic Detection

We performed background subtraction by calculating a linearly weighted average of 50 images via the “bsubtract” module of the Gtrack machine vision kit (Borg Lab, http://borg.cc.gatech.edu; see also http://www.bio-tracking.org). This enabled us to differentiate changing areas of the image (flies) from static components and simplify the visual data.

We then used threshold-based segmentation in ImageJ [27], via a custom macro script. This script enabled us to detect individual flies based on the size of contiguous segmented blobs. The script would automatically open a stack of images from a single camera during one replicate, convert these to 8-bit images and then threshold them to differentiate insects from background. The pixels above threshold were “blobbed” (to avoid overcount) via binary “open” and “close” procedures. Finally, we used the built-in “Analyze Particles” function to detect flies with a total area of 150–950 pixels. Further details on the procedure and scripts can be obtained at http://unitsci.org:8080/vis/IL_vid/index.html, and from a previous publication that employed the same method [28].

## Results

### Validation

In order to check the accuracy of the automatic counts we selected ≈ 20 images from each camera per date (240 images per experiment day) and manually counted the number of flies visible on the trap surface pictured. This resulted in a total validation dataset of 1730 images, which we used to compare the number of flies automatically detected with the number counted manually. We found that in 79% of the validation images the automatic count matched the manual count or was off by one. Ninety-one percent of the validation images had the automatic and manual counts within 3 individuals. The automatic counts were generally lower than the actual number in the image.

**Fig 2:**
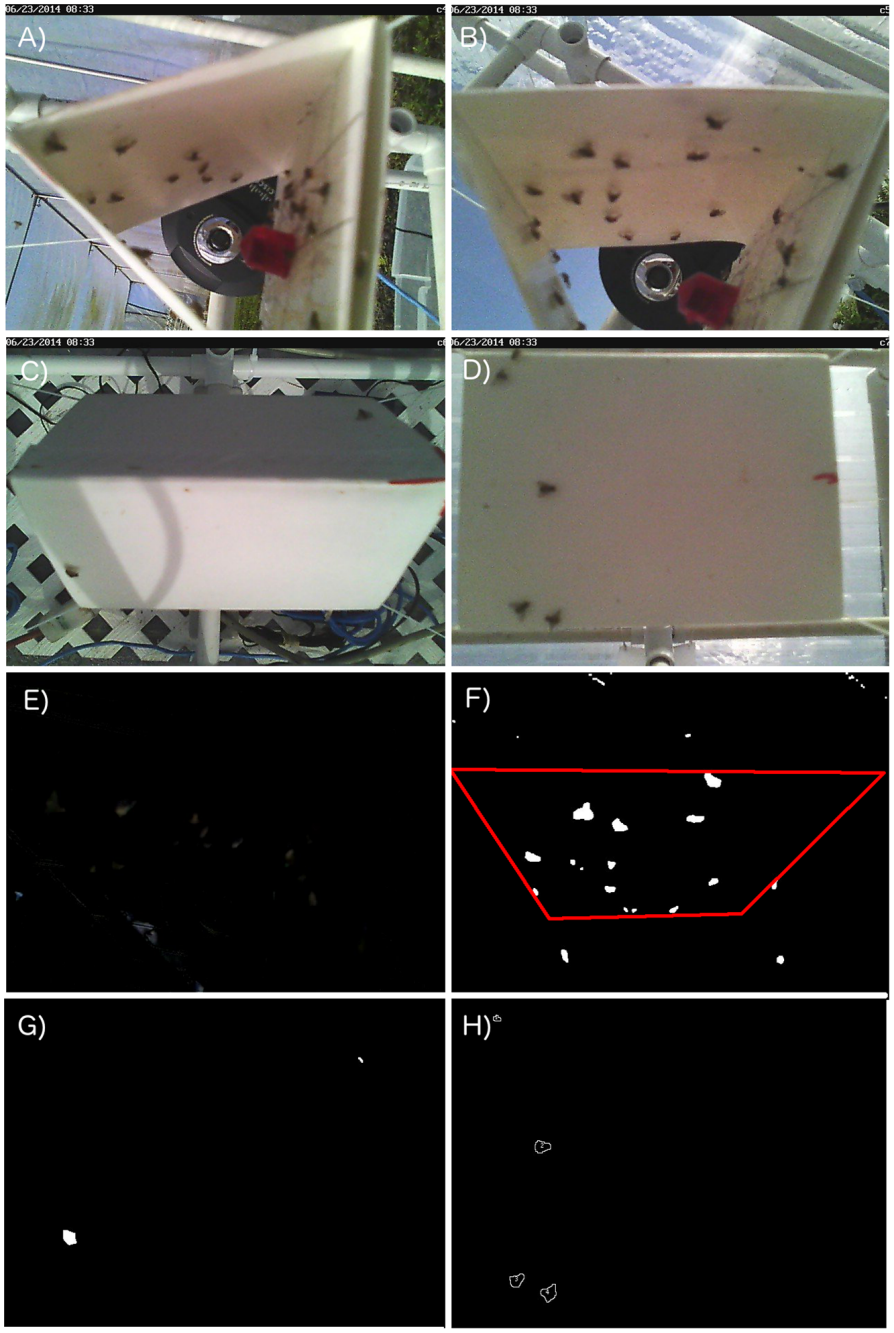
Sample raw and processed images. Raw images captured at 08:33 on 23 June 2014 of a Jackson trap baited with a TML plug and no insecticide: A) Internal, East; B) Internal, West; C) Top, West and East; D) Bottom. Processed images: E) Image from A after background subtraction; F) Image from B after background subtraction and segmentation (region of interest marked in red); G) Image from C after background subtraction and segmentation; H) Image from D after background subtraction, segmentation and automated detection (outlines of detected flies are marked and numbered).

To further compare the automatically detected and manually counted number of flies we calculated correlations between these two variables. For all experimental dates, manual and automatic counts had a Pearson correlation coefficient (*r*) of 0.81. *r* for each date individually ranged from 0.72 to 0.89. All correlations were statistically significant at *α* = 0.001. Sample images are shown in Fig 2.

### Detections on Trap Surfaces

We automatically detected the number of flies on 381,957 images of trap surfaces across all experiments. The average number of flies detected on all monitored surfaces of the TML-DDVP baited traps per set of images (all images of a given trap at one time step) was 4.15 ±0.39 (mean ± SD). This was half the number detected on TML traps (8.30 ±1.18). For the control traps, the average number of flies automatically detected on all surfaces was 0.76 ±0.08 per image set.

A two-way analysis of variance (ANOVA) was used to analyze the effect of bait type and surface on the number of flies automatically detected. The ANOVA also included an interaction between the two predictor variables. The response variable was log-transformed to ensure normality (Kolmogorov-Smirnov 1-way test of the transformed response against a normal distribution with the observed mean and standard deviation: *D* = 0.095, *p* = 0.305). This test showed a significant effect of both factors but not their interaction at *α* = 0.05 (Table 1).

**Table 1:** Two-way ANOVA of the effect of bait type and surface on the number of flies observed. Levels of factor “Bait” are Control, TML, and TML-DDVP. For “Surface”, levels are Internal East, Internal West, Top East, Top West, and Bottom. Response variable is the number of flies automatically detected (log transformed).

Factor	df	Sum of squares	Mean Square	*F* value	*p* value
Bait	2	121.2	60.6	93.6	<0.001
Surface	4	21.2	5.3	8.2	<0.001
Bait x Surface	8	9.9	1.2	1.9	0.069
Residuals	88	57.0	0.6		

**Fig 3:**
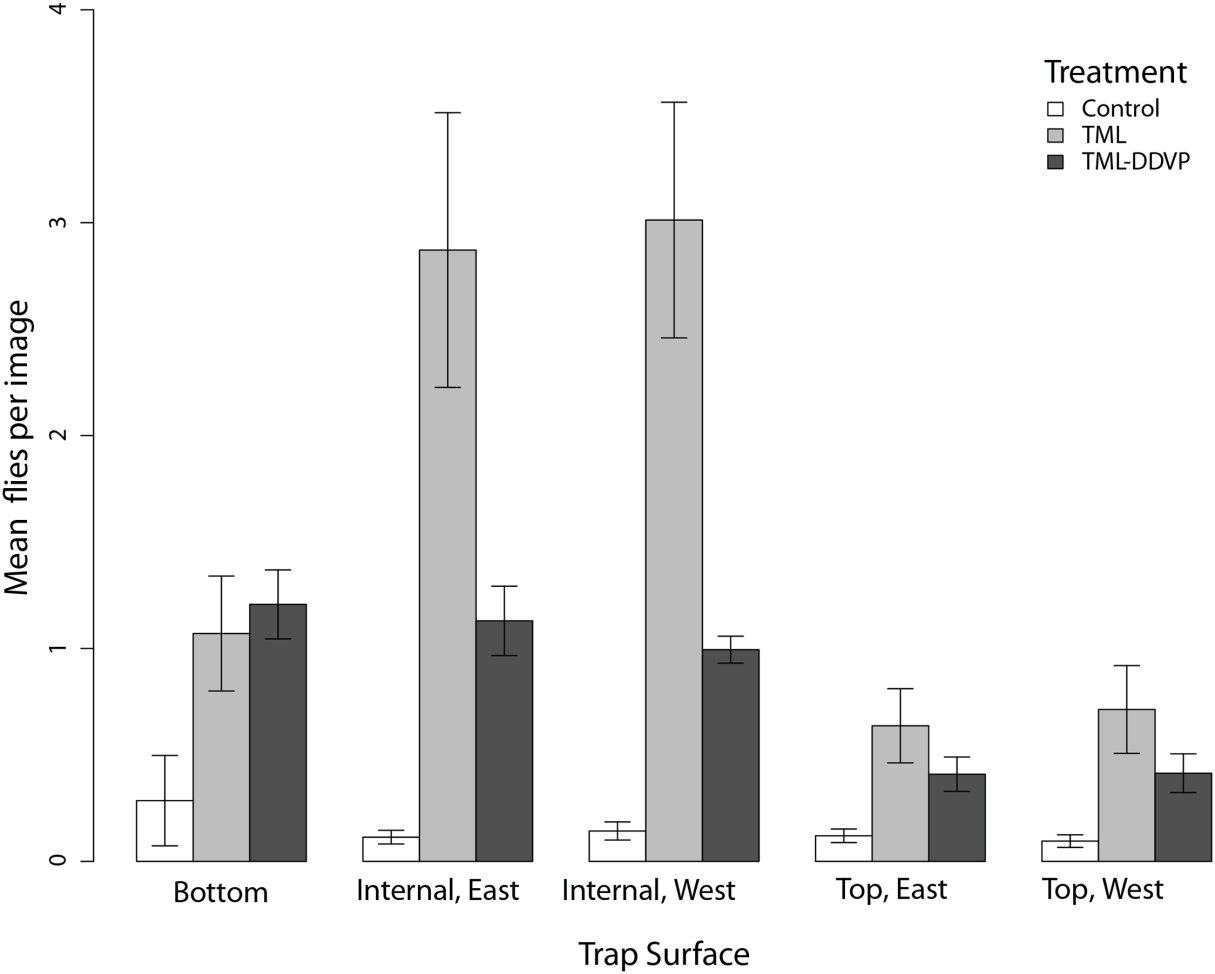
Average and SE of the number of flies automatically detected on each monitored surface of the Jackson traps by treatment. The mean number of flies per image was statistically significantly different for the internal panels. See text for more details.

The average number of flies per surface by treatment can be seen in Fig 3. Post hoc comparisons of each surface between TML and TML-DDVP treatments via Fisher’s Least Significant Difference (LSD) test showed significantly more detections in the Internal East and Internal West panels of the TML traps compared with the same for the TML-DDVP traps. All other comparisons (Top East, Top West and Bottom) were not significantly different between TML and TML-DDVP. All control trap surfaces had significantly lower detections compared with equivalent surfaces from both TML and TML-DDVP.

### Captures

The number of flies captured in traps and dead individuals found in tubs under the traps per experimental day and by treatment are given in Table 2. For all tests in this section transformed data were checked for normality via Kolmogorov-Smirnov tests and analyzed via one-way ANOVA. ANOVAs were used to compare the number of flies caught on the sticky panels of traps baited with TML with those baited with TML-DDVP, as well as the number caught in the tubs below each trap and the sum of the number in the trap and in the tub (“total”) for these traps. The total number of adult medflies (trapped + in tub) was similar between TML and TML-DDVP treatments (*F*_1,12_ = 1.50, *p* = 0.245), just as the number caught in the sticky panels of the traps alone (*F*_1,12_ = 0.43, *p* = 0.525). However, the number of dead flies found in the tub was significantly higher for the TML-DDVP traps compared with the TML traps (*F*_1,12_ = 6.73, *p* = 0.024).

**Table 2:** Number of flies captured in traps and found in tub per treatment.

		TML			TML-DDVP			Control		
Date	Released	Trapped	In Tub	Total	Trapped	In Tub	Total	Trapped	In Tub	Total
23 May 2014	1636	291	57	348	287	31	318	26	2	28
30 May 2014	903	294	42	336	235	79	314	134	3	137
16 June 2014	1488	401	52	453	381	75	456	13	1	14
23 June 2014	1172	163	4	167	388	69	457	11	1	12
07 July 2014	1217	292	26	318	410	153	563	17	0	17
14 July 2014	1712	244	10	254	543	178	721	47	1	48
18 July 2014	1674	584	52	636	249	56	305	107	6	113
**Mean±SD**	1400.3±307.8	324.1±134.7	34.7±21.5	358.9±150.4	356.1±108.3	91.6±53.4	447.7±154.5	50.7±49.8	2.0±2.0	52.7±51.3

## Discussion

The computer vision results reveal that the majority of fly sightings occurred in the two inner panels compared with the three outer surfaces of the Jackson trap in the absence of DDVP (71% of total sightings for TML only traps were on inner panels). With DDVP, inner panel sightings were significantly lower, probably because of mortality rather than repellence (since catches were equivalent). Mortality from the insecticide plus multiple sightings of the same individual on images account for the difference between the CV results and the capture counts. Adding sticky surfaces inside the Jackson trap should increase its efficiency, but an even better alternative might be to add a contact insecticide to the inner panels. This would ensure that flies that landed or walked on the inside of the trap would likely be killed and drop on to the sticky panel for capture.

Before this study, the effect of adding an insecticide such as DDVP to a TML-baited trap has been examined by comparing the total catch after a given period of time. Using only trap catch data, results on the effect of DDVP have been contradictory. Some researchers indicate reduced captures with insecticide, and cite a repellent effect of DDVP on *C. capitata*[18, 20]. However, other data find no difference in captures: Kasoyannos et al [29] did not find an effect of DDVP on trap captures using McPhail traps, and neither did Wijesuriya and De Lima using Jackson traps [30]. In this study, we did not find a difference in the number of Medfly captured on the sticky panels of TML-baited Jackson traps with and without DDVP.

The effect of DDVP on trap catches has been examined in more detail for *Bactrocera* fruit flies, where the possibility of repellence by DDVP has also been suggested [31], though others have found higher trap captures with DDVP than without [19, 32] or no significant difference [33]. Where weekly trap counts have been made, data indicate that including a DDVP vapor strip can lead to initially depressed catches (for about the first week) and then captures return to levels comparable to those from traps without DDVP [21]. This pattern conforms to the known rapid decrease of DDVP vapors in the field [32], and reinforces the suggestion that flies are attracted to the lure normally in the presence of DDVP, but perish more frequently before entering the trap, resulting in decreased trap capture efficiency [20, 21]. Build-up of insecticide fumes around traps with DDVP has been measured [34], which is probably responsible for the decrease in trap capture efficiency. In this case, reducing the dose of DDVP could reduce the problem of initial trap inefficiency, leading to better capture efficiency for *Bactrocera* fruit flies.

Our observation of a significantly larger number of flies in the tubs under the traps with DDVP compared with the TML- only traps supports the idea of possible decreased trap efficiency when DDVP is used with TML to capture Medflies. Repellence by DDVP, causing some flies to not enter the traps and instead be killed outside, seems unlikely given the equivalence in the number trapped. Further, the similar number of flies sighted on the top and bottom panels of the TML- DDVP and TML only traps supports no repellent effect of DDVP on Medflies.

In our results the proportion of total flies found in tubs relative to the number caught in traps was higher with DDVP compared to TML alone (Table 2). In a low population density situation, such as under detection monitoring, there might be decreased probability of capturing a fly approaching the trap with DDVP. This would apply if any of the flies around the trap were destined to eventually enter it and be captured, which is not suggested by the equivalence in actual trap catches in this study. It is possible that many of the insects counted in the tubs were females. In any case, it remains reasonable to rely on trapping without insecticide for *C. capitata* under detection, currently the standard procedure in California [8].

The results from this experiment should be interpreted with caution because we did not produce separate counts of males and females sighted, trapped and caught in the tubs. We expect the majority of flies coming to the traps to be males, as females are only attracted when low numbers of males are present [35]. However, females are known to aggregate around TML-baited traps, perhaps using these as lekking sites [36–38].

## Supporting Information

S1 File Automatic detection data, validation and processing macros. Validation and automatic detection data are in separate plain text comma-separated-value (csv) files by date. Macros can be run in ImageJ 1.45b and contain settings used for automatic detection on each date.
(ZIP)
